# Supplementary material for: Resolving the full spectrum of human genome variation using Linked-Reads
Source: Genome Res. 2019 Apr;29(4):635–45. doi: 10.1101/gr.234443.118 (PMC6442396; doi:10.1101/gr.234443.118)
Supplement: Supplemental Material [file supp_gr.234443.118_Supplemental_longranger-2.2.zip › longranger-2.2.tar.bz2!/lr2.2-public/lib/stan/cmdstan/stan/doc/api/html/math_2matrix_2mdivide__left__tri_8hpp_source.html]

Stan: src/stan/math/matrix/mdivide\_left\_tri.hpp Source File


|  |  |
| --- | --- |
|  | Stan  2.5.0  probability, sampling & optimization |


- Main Page
- Related Pages
- Namespaces
- Classes
- Files

- File List
- File Members

All Classes Namespaces Files Functions Variables Typedefs Enumerations Enumerator Friends Macros Pages

- src
- stan
- math
- matrix

mdivide\_left\_tri.hpp

Go to the documentation of this file.

1 #ifndef STAN\_\_MATH\_\_MATRIX\_\_MDIVIDE\_LEFT\_TRI\_HPP

2 #define STAN\_\_MATH\_\_MATRIX\_\_MDIVIDE\_LEFT\_TRI\_HPP

3

4 #include <boost/math/tools/promotion.hpp>

5 #include <stan/math/matrix/Eigen.hpp>

6 #include <stan/math/matrix/promote\_common.hpp>

7 #include <stan/math/error\_handling/matrix/check\_multiplicable.hpp>

8 #include <stan/math/error\_handling/matrix/check\_square.hpp>

9

10 namespace stan {

11  namespace math {

12

22  template <int TriView, typename T1, typename T2,

23  int R1, int C1, int R2, int C2>

24  inline

25  Eigen::Matrix<typename boost::math::tools::promote\_args<T1,T2>::type,

26  R1,C2>

27  mdivide\_left\_tri(const Eigen::Matrix<T1,R1,C1> &A,

28  const Eigen::Matrix<T2,R2,C2> &b) {

29  stan::math::check\_square("mdivide\_left\_tri(%1%)",A,"A",(double\*)0);

30  stan::math::check\_multiplicable("mdivide\_left\_tri(%1%)",A,"A",

31  b,"b",(double\*)0);

32  return promote\_common<Eigen::Matrix<T1,R1,C1>,Eigen::Matrix<T2,R1,C1> >(A)

33  .template triangularView<TriView>()

34  .solve( promote\_common<Eigen::Matrix<T1,R2,C2>,

35  Eigen::Matrix<T2,R2,C2> >(b) );

36  }

37

45  template<int TriView, typename T,int R1, int C1>

46  inline

47  Eigen::Matrix<T,R1,C1>

48  mdivide\_left\_tri(const Eigen::Matrix<T,R1,C1> &A) {

49  stan::math::check\_square("mdivide\_left\_tri(%1%)",A,"A",(double\*)0);

50  int n = A.rows();

51  Eigen::Matrix<T,Eigen::Dynamic,Eigen::Dynamic> b;

52  b.setIdentity(n,n);

53  A.template triangularView<TriView>().solveInPlace(b);

54  return b;

55  }

56

57  }

58 }

59 #endif

check\_multiplicable.hpp

promote\_common.hpp

stan::math::check\_multiplicable

bool check\_multiplicable(const char \*function, const T1 &y1, const char \*name1, const T2 &y2, const char \*name2, T\_result \*result)

**Definition:** check\_multiplicable.hpp:15

Eigen.hpp

stan::math::promote\_common

common\_type< T1, T2 >::type promote\_common(const F &u)

**Definition:** promote\_common.hpp:14

stan::math::mdivide\_left\_tri

Eigen::Matrix< typename boost::math::tools::promote\_args< T1, T2 >::type, R1, C2 > mdivide\_left\_tri(const Eigen::Matrix< T1, R1, C1 > &A, const Eigen::Matrix< T2, R2, C2 > &b)

Returns the solution of the system Ax=b when A is triangular.

**Definition:** mdivide\_left\_tri.hpp:27

stan::math::check\_square

bool check\_square(const char \*function, const Eigen::Matrix< T\_y, Eigen::Dynamic, Eigen::Dynamic > &y, const char \*name, T\_result \*result)

Return true if the specified matrix is square.

**Definition:** check\_square.hpp:24

check\_square.hpp


---

[ Stan Home Page ]

*© 2011–2014,
Stan Development Team.*
